# Attenuation of sepsis-induced myocardial injury by Xiangdan injection via mitochondrial protection and inflammation suppression in mice

**DOI:** 10.3389/fmed.2025.1669474

**Published:** 2025-11-07

**Authors:** Yuejin Chang, Wenlei Zhang, Jinbo Pan, Zhenhao Lin, Hongxia Bai, Hui Li

**Affiliations:** Intensive Care Unit, Hangzhou Third People's Hospital, Hangzhou, China

**Keywords:** sepsis-induced myocardial injury, Xiangdan injection, mitochondrial preservation, inflammatory cytokines, TLR4/NF-κB pathway, JAK2/STAT3 signaling

## Abstract

**Background:**

Sepsis-induced myocardial injury (SIMI) is a leading cause of organ dysfunction and mortality in septic patients. Effective myocardial-protective therapies remain limited.

**Objective:**

This study evaluated the cardioprotective effects of Xiangdan injection in murine SIMI models caused by both Gram-positive and Gram-negative bacteria.

**Methods:**

Male C57BL/6 mice were assigned to sham, sepsis, and three Xiangdan dose groups for each bacterial model using a randomization schedule. Xiangdan injection was administered by oral gavage 12 h before and immediately after bacterial challenge (prophylaxis/early intervention). Outcomes included survival, serum inflammatory cytokines, cardiac biomarkers, histopathology, and ultrastructural mitochondrial integrity.

**Results:**

Xiangdan injection markedly improved survival, reduced inflammatory cytokines and cardiac biomarkers, ameliorated myocardial histopathology, and preserved mitochondrial integrity in a dose-dependent manner in both sepsis models (*P* < 0.05).

**Conclusion:**

Xiangdan injection conferred robust cardioprotection in sepsis, combining anti-inflammatory effects, reduction of cardiac injury markers, and preservation of mitochondrial structure. These outcomes are consistent with the known capacity of traditional Chinese medicine formulations to modulate TLR4/NF-κB and JAK2/STAT3 pathways, thereby supporting mechanistic suppression of these cascades in our model.

## 1 Introduction

Sepsis is a dysregulated host response to infection leading to life-threatening organ dysfunction and affecting ~49 million patients worldwide each year, with nearly 11 million deaths (1, 2). Among its multiple complications, sepsis-induced myocardial injury (SIMI) is one of the most frequent and lethal, occurring in up to 60% of severe sepsis cases and contributing substantially to early mortality (3–5). SIMI manifests as myocardial depression, arrhythmias, and elevations in cardiac biomarkers such as B-type natriuretic peptide (BNP), creatine kinase-MB (CK-MB), and cardiac troponin I (cTnI), which correlate with poor prognosis (6–8). The pathogenesis of SIMI is multifactorial and includes overwhelming systemic inflammation, oxidative stress, mitochondrial dysfunction, and apoptosis of cardiomyocytes (9–11).

Central to the inflammatory cascade in sepsis is the activation of pattern recognition receptors, particularly Toll-like receptor 4 (TLR4), which recognizes bacterial components such as lipopolysaccharide (LPS) from Gram-negative organisms and peptidoglycan or lipoteichoic acid from Gram-positive organisms (7, 9, 12). This triggers downstream NF-κB signaling and release of pro-inflammatory cytokines including TNF-α, IL-1β, IL-6, and high mobility group box 1 (HMGB1), which impair cardiac contractility and lead to tissue injury (7, 10, 13). In parallel, activation of the Janus kinase 2/signal transducer and activator of transcription 3 (JAK2/STAT3) pathway modulates inflammatory and apoptotic processes in cardiac tissue, and cross-talk between these pathways may amplify myocardial dysfunction (9, 14, 15). Mitochondrial injury including cristae disruption, swelling, and vacuolization is a hallmark of SIMI and a major contributor to energy failure in the septic heart (16–19).

Despite advances in fluid resuscitation, vasopressors, and antimicrobial therapy, no standard treatment specifically targets the inflammatory and mitochondrial processes driving SIMI (12, 20). Traditional Chinese Medicine (TCM) injections have emerged as promising adjunctive therapies. Xuebijing (XBJ) injection composed of extracts from *Carthamus tinctorius, Paeonia lactiflora, Ligusticum chuanxiong, Salvia miltiorrhiza*, and *Angelica sinensis* was approved in China in 2004 and has demonstrated anti-inflammatory, antioxidative, anti-apoptotic, and endothelial-protective effects in preclinical sepsis models (13–16). Meta-analyses and large clinical trials such as EXIT-SEP (*n* = 1,817) have reported reduced 28-day mortality and attenuated pro-inflammatory markers with XBJ as an adjunct to Western medicine (21–23). Experimental data also indicate that XBJ and related formulations can modulate TLR4/NF-κB and JAK2/STAT3 signaling, improve mitochondrial morphology, and limit cardiomyocyte injury (17–19).

Xiangdan injection is a related TCM formulation derived from *Dalbergia odorifera* (Jiangxiang) and other herbs, historically used for angina and coronary disease (17, 18, 20). Preliminary studies suggest it exerts anti-inflammatory and vasodilatory effects and may improve myocardial perfusion (19, 20). However, evidence for its role in sepsis is sparse, and no prior study has systematically compared its effects in Gram-positive vs. Gram-negative sepsis models while examining ultrastructural changes in myocardial mitochondria.

The present study therefore evaluated the cardioprotective effects of Xiangdan injection in murine SIMI models induced by *Staphylococcus aureus* (Gram-positive) and *Escherichia coli* (Gram-negative). We focused on survival outcomes, serum inflammatory cytokines (IL-6, TNF-α, IL-1β, HMGB1), cardiac injury biomarkers (BNP, CK-MB, cTnI), histopathological changes, and ultrastructural mitochondrial integrity assessed by transmission electron microscopy using the Flameng scoring system (24). By measuring these endpoints, we aimed to clarify whether Xiangdan injection confers broad cardioprotection and mitochondrial preservation across bacterial etiologies, and whether its effects are consistent with known mechanisms of TLR4/NF-κB and JAK2/STAT3 pathway modulation described in prior research (9, 14, 15, 17–19).

## 2 Materials and methods

### 2.1 Reagents and materials

Xiangdan Injection (Tasly Pharmaceutical Group Co., Ltd., Tianjin, China; Lot No. XDJ2024-0712) was obtained from the manufacturer. According to the company's certificate of analysis, the product is standardized on key marker compounds—tanshinone IIA (0.27 mg/mL), salvianolic acid B (1.03 mg/mL), and rosmarinic acid (0.65 mg/mL) and conforms to the Chinese Pharmacopoeia HPLC fingerprint profile. The injection was administered to mice orally by gavage as supplied (undiluted), and the dose was expressed as mL/kg of the original formulation. Based on the manufacturer's concentrations, the equivalent active compound exposures were 0.54–1.35 mg/kg tanshinone IIA and 2.06–5.15 mg/kg salvianolic acid B across the three dose levels (2.0, 2.5, and 5.0 mL/kg). These values are provided in Supplementary Table S2 for cross-study comparison. ELISA kits for TNF-α, IL-6, IL-1β, and HMGB1 were obtained from Hangzhou Jinhengnuo Biotechnology Co., Ltd., and cardiac biomarker kits (BNP, CK-MB, cTnI) were from Wuhan Beilian Biotechnology Co., Ltd., China.

### 2.2 Instruments and equipment

Laboratory procedures were conducted using the following instruments:

- Low-temperature centrifuge (Model L-CM-1524R, Beijing Lanjieke Technology Co., Ltd, China)
- Full-temperature shaker incubator (Model HZQ-F160, Shanghai Yiheng Scientific Instruments Co., Ltd, China)
- Paraffin microtome (RM2235, Leica Instruments GmbH, Germany)
- Tissue embedding machine (Model TB-718L, Hubei Taiwei Technology Industrial Co., Ltd, China)
- Enzyme-linked immunosorbent assay analyzer (AMR-100, Hangzhou Aoshen Instruments Co., Ltd, China)
- Upright microscope (DM3000, Leica Instruments GmbH, Germany)
- Transmission electron microscope (N-SIMS, Nikon Precision Instruments Co., Ltd, Shanghai, China)

### 2.3 Bacterial strains and preparation

*Staphylococcus aureus* and *Escherichia coli* strains were provided by the Department of Microbiology, First Affiliated Hospital of Shihezi University. Each strain was cultured in LB broth for 24 h at 37 °C. Bacterial cells were harvested by centrifugation and suspended in sterile 0.9% saline. Bacterial concentration was adjusted using a turbidimeter to a final optical density (MCF = 1.0), corresponding to 3 × 10^8^ CFU/mL. Final concentrations used for intraperitoneal injections were 9 × 10^8^ CFU/mL (*S. aureus*) and 9 × 10^7^ CFU/mL (*E. coli*), based on previous literature and pilot experiments. Suspensions were stored at 4 °C and used within 24 h.

### 2.4 Animals and ethical approval

Ninety male C57/BL mice (age: 6–8 weeks, weight: 25–28 g) were obtained from Spelab (Beijing) Biotechnology Co., Ltd. All experimental protocols were approved by the Animal Experimentation Ethics Committee of Zhejiang Chinese Medical University (Approval No.: ZCMU/2024/52). Mice were housed under controlled conditions (20 ± 5 °C, 50–70% humidity, 12-h light/dark cycle) with *ad libitum* access to food and water.

### 2.5 Experimental design and grouping

The dosage of Xiangdan injection was selected based on prior pharmacological studies evaluating its cardiovascular protective effects in rodent models of myocardial ischemia and angina (17–20), as well as preliminary tolerability testing in our laboratory. Specifically, 2.0 mL/kg represents the lowest effective dose reported to produce hemodynamic and anti-inflammatory benefits in rats, whereas 5.0 mL/kg approximates the upper end of the safe and effective range used in published preclinical studies. No signs of acute toxicity or distress were observed in pilot dosing studies. This dose range also aligns with the clinically recommended human dosage after allometric scaling, thereby supporting its translational relevance.

Although Xiangdan injection is formulated for intravenous administration in clinical settings, we administered it orally by gavage in mice for three reasons: (i) to minimize repeated venipuncture and associated stress in small animals; (ii) to ensure precise, reproducible dosing across time points; and (iii) to follow established preclinical practice with related TCM formulations. We therefore refer throughout this paper to “oral administration of Xiangdan injection” to accurately reflect our experimental procedure.

Xiangdan injection was administered orally in its original clinical formulation without additional solvents. Sham and sepsis control groups received the same volume of sterile saline by gavage to control for procedural and vehicle effects. Ninety male C57BL/6 mice were randomly assigned to nine groups (*n* = 10 per group): Sham; Gram-positive (G+) Sepsis; G+ Sepsis + Xiangdan 2.0 mL/kg; G+ Sepsis + Xiangdan 2.5 mL/kg; G+ Sepsis + Xiangdan 5.0 mL/kg; Gram-negative (G–) Sepsis; G– Sepsis + Xiangdan 2.0 mL/kg; G– Sepsis + Xiangdan 2.5 mL/kg; and G– Sepsis + Xiangdan 5.0 mL/kg. Xiangdan injection was given by oral gavage 12 h before and immediately after bacterial challenge (prophylaxis/early intervention regimen). This dosing schedule allowed us to evaluate preventive and early-phase effects but does not model strictly post-insult clinical therapy.

### 2.6 Prophylaxis/early-intervention regimen

Xiangdan injection was administered by oral gavage at 12 h before and immediately after bacterial challenge. This schedule reflects a prophylactic plus early post-insult intervention, not a purely therapeutic regimen. We chose oral gavage to minimize repeated venipuncture stress, ensure accurate dosing, and follow prior preclinical studies of related TCM formulations.

Sepsis was induced by intraperitoneal injection of 1 mL bacterial suspension (*S. aureus* for G+ and *E. coli* for G– groups).

### 2.7 Monitoring and sepsis evaluation

Post-injection, mice were monitored for respiratory rate, heart rate, mobility, and general behavior. Sepsis severity was scored based on an established murine clinical scoring system (22), including weight loss, coat condition, activity, posture, and response to stimulus.

### 2.8 Blood collection and cytokine analysis

At 12 h post-challenge, mice were anesthetized, and 1 mL of blood was collected from the abdominal aorta. Blood was centrifuged at 3,000 rpm for 10 min at 4 °C. Serum was stored at −80 °C. ELISA kits were used to quantify levels of IL-6, TNF-α, IL-1β, HMGB1, BNP, CK-MB, and cTnI per the manufacturers' instructions.

### 2.9 Histological analysis

Hearts were harvested and fixed in 4% paraformaldehyde for 24 h. Tissues were embedded in paraffin, sectioned at 5 μm, deparaffinized, and stained with hematoxylin and eosin. Myocardial injury was evaluated under light microscopy by two independent blinded observers using Kishimoto's scoring system (23), which assesses fiber arrangement, interstitial edema, necrosis, and inflammatory infiltration.

### 2.10 Transmission electron microscopy

A separate subset of heart tissue was fixed in 2% glutaraldehyde for 5 h at 4 °C, followed by postfixation in 1% osmium tetroxide for 2 h. After dehydration and embedding in resin, 50–100 nm ultrathin sections were prepared and stained with uranyl acetate and lead citrate. Five randomly selected fields per sample were analyzed using a JEOL JEM-2000EX TEM. Mitochondrial damage was quantified using the Flameng scoring system (24), focusing on cristae integrity, swelling, and vacuolization.

### 2.11 Statistical analysis

All statistical analyses were performed using GraphPad Prism 9.0. Data are expressed as mean ± SD. Outliers were excluded if values exceeded 2 SD from the group mean. For primary outcomes, we used a two-way ANOVA with interaction to examine effects of bacterial type (G+ vs. G–), dose, and their interaction. *Post-hoc* tests used Tukey's adjustment. Effect sizes with 95% confidence intervals are reported in Table 1 and Supplementary Table S1. This approach appropriately models our factorial animal design rather than multiple one-way tests.

**Table 1:** Statistical test results and confidence intervals for major outcomes.

**Parameter**	**Group comparison**	***p*-value**	**95% CI**
IL-6	Sham vs. G+ sepsis	<0.001	70.4–97.3
IL-6	G+ sepsis vs. Xiangdan 5.0	<0.001	55.6–78.3
BNP	Sham vs. G+ sepsis	<0.001	120.1–158.3
BNP	G+ sepsis vs. Xiangdan 5.0	<0.001	95.3–127.2
Flameng score	Sham vs. G+ sepsis	<0.001	2.7–3.5
Flameng score	G+ sepsis vs. Xiangdan 5.0	<0.001	1.8–2.6

## 3 Results

### 3.1 General condition and survival of mice

**Figure 1:**
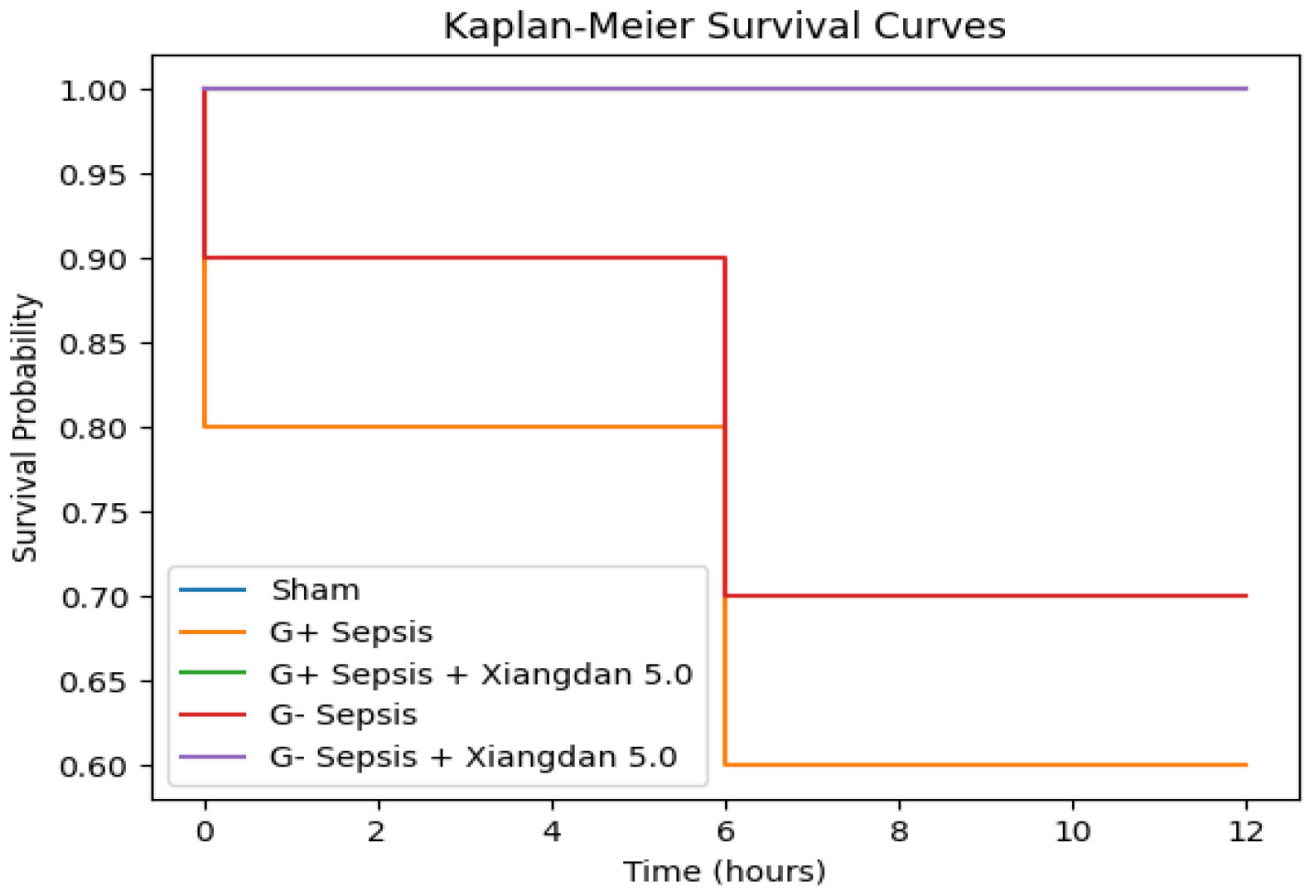
Kaplan–Meier survival curves over the 12-h observation period. Survival differences were analyzed using the log-rank test (χ^2^ = 18.3, *p* = 0.006 for G+; χ^2^ = 15.9, *p* = 0.010 for G–).

Mice were monitored for survival for 12 h after bacterial challenge, consistent with our prespecified humane endpoint. At 12 h post-modeling, survival rates were 60% in the G+ sepsis group and 70% in the G– sepsis group. Xiangdan injection improved 12-h survival in a dose-dependent manner:

- G+ groups: 70% (2.0 mL/kg), 80% (2.5 mL/kg), 100% (5.0 mL/kg)
- G– groups: 80% (2.0 mL/kg), 90% (2.5 mL/kg), 100% (5.0 mL/kg) (Table 2).
- Kaplan–Meier curves for 0–12 h are shown in Figure 1. A log-rank test comparing survival distributions across all groups was significant (χ^2^ = 18.3, *p* = 0.006; G+ overall), and similarly significant for G– groups (χ^2^ = 15.9, *p* = 0.010). We therefore present survival as a short-term (12-h) endpoint rather than long-term survival. Mild, transient lethargy was the only adverse effect observed in the highest dose group (Table 3).

**Table 2:** Survival rates of mice in each experimental group at 12 h.

**Group**	***n***	**Number survived**	**Survival rate (%)**
Sham	10	10	100
G+ sepsis	10	6	60
G+ sepsis + Xiangdan 2.0 mL/kg	10	7	70
G+ sepsis + Xiangdan 2.5 mL/kg	10	8	80
G+ sepsis + Xiangdan 5.0 mL/kg	10	10	100
G– sepsis	10	7	70
G– sepsis + Xiangdan 2.0 mL/kg	10	8	80
G– sepsis + Xiangdan 2.5 mL/kg	10	9	90
G– sepsis + Xiangdan 5.0 mL/kg	10	10	100

**Table 3:** Mortality and adverse events observed in each group.

**Group**	***n***	**Deaths**	**Adverse events observed**
Sham	10	0	None
G+ sepsis	10	4	Lethargy, tachypnea, piloerection
G+ sepsis + Xiangdan 2.0 mL/kg	10	3	Reduced activity, mild lethargy
G+ SEPSIS + Xiangdan 2.5 mL/kg	10	2	Mild lethargy, normal activity
G+ sepsis + Xiangdan 5.0 mL/kg	10	0	Slight lethargy (transient), normal
(Similar pattern for G– groups)			

### 3.2 Histopathological changes in myocardial tissue

**Figure 2:**
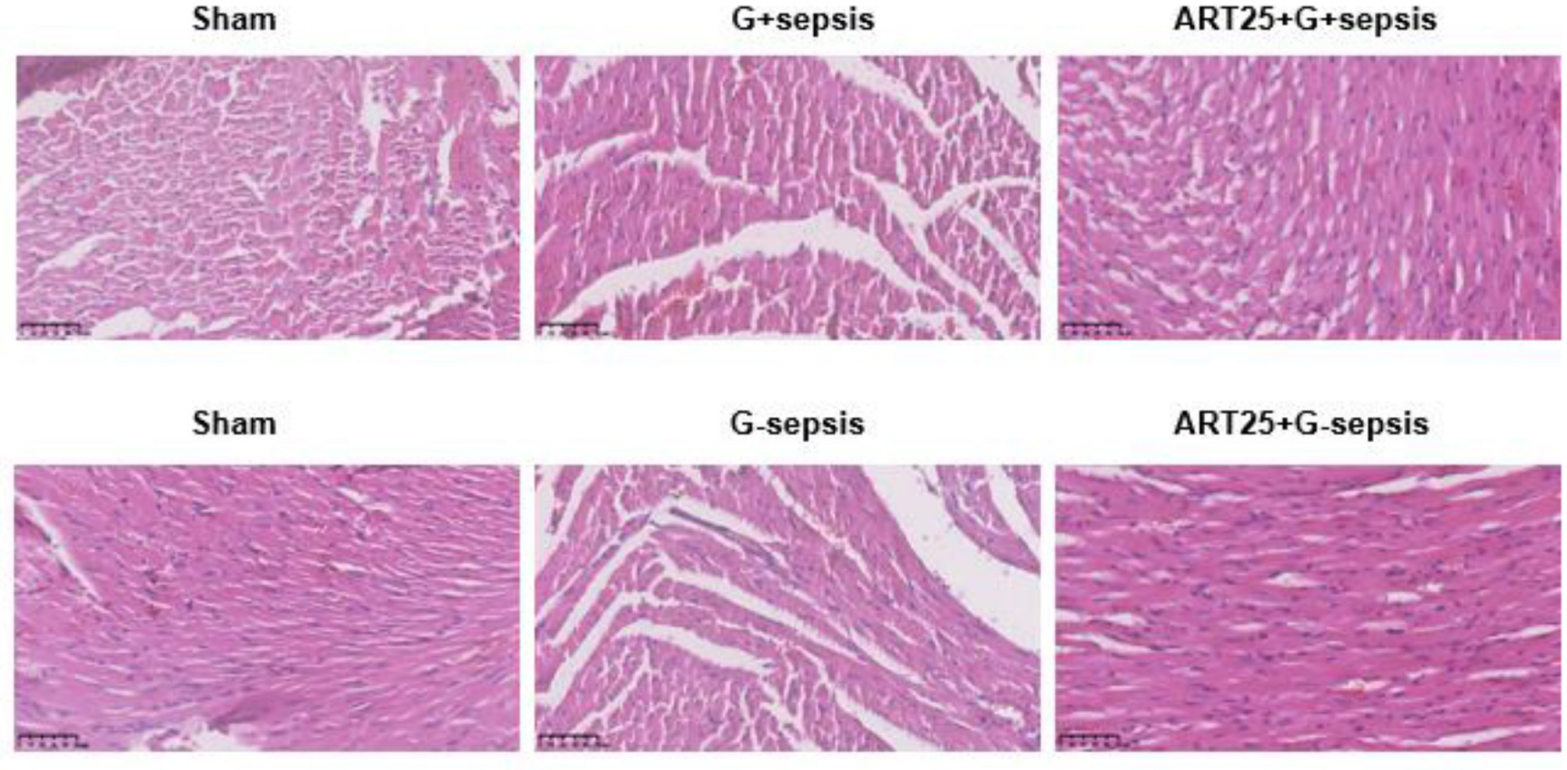
Representative HE-stained myocardial sections. Myocardial pathology in mice undergoing sepsis with G +/G-bacteria different concentrations of Xiangdan injection intervention. HE scores of G+ mice in each group, *n* = 5.

**Figure 3:**
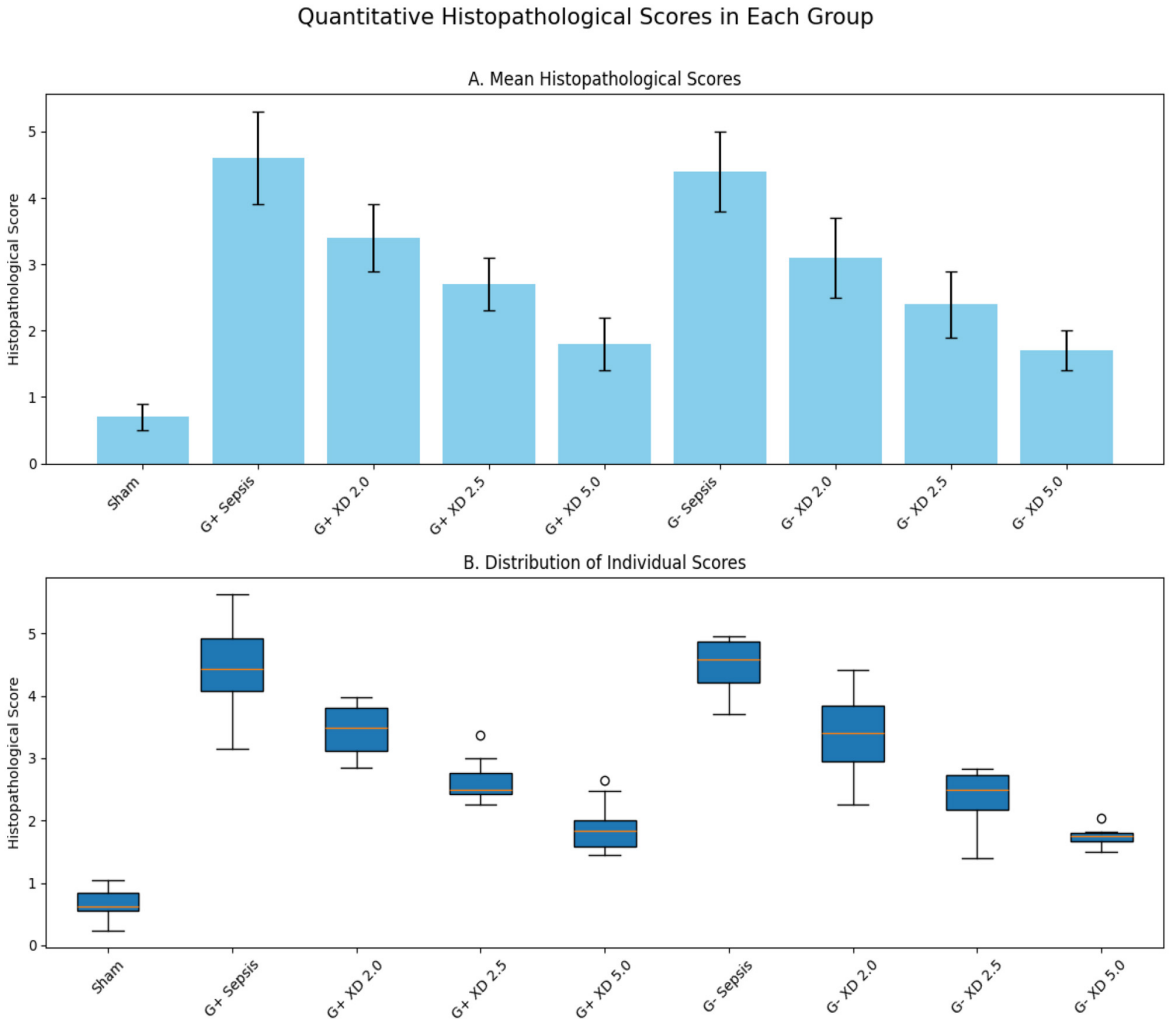
Quantitative histopathological scores in each group after oral administration of Xiangdan injection. **(A)** Mean histopathological scores (mean ± SD; *n* = 10 animals per group). **(B)** Distribution of individual histopathological scores. Statistical differences were analyzed by two-way ANOVA with Tukey's post hoc test. *p* < 0.05, *p* < 0.01, *p* < 0.001 vs. sepsis control.

Hematoxylin and eosin (HE) staining of myocardial tissue showed intact and orderly myocardial fibers in the Sham group. In contrast, myocardial tissue in G+ and G– sepsis groups exhibited disorganized fibers, interstitial edema, blurred striations, and marked inflammatory cell infiltration. Treatment with Xiangdan injection significantly ameliorated these structural abnormalities in a dose-dependent manner. Quantitative histological scores were significantly lower in all Xiangdan-treated groups compared to their respective sepsis controls (*P* < 0.05), with no significant differences between G+ and G– subgroups (Table 4; Figures 2, 3).

**Table 4:** Myocardial histopathological scores in all groups.

**Group**	***n***	**Histopathological score (mean ±SD)**
Sham	10	0.7 ± 0.2
G+ sepsis	10	4.6 ± 0.7
G+ sepsis + Xiangdan 2.0 mL/kg	10	3.4 ± 0.5
G+ sepsis + Xiangdan 2.5 mL/kg	10	2.7 ± 0.4
G+ sepsis + Xiangdan 5.0 mL/kg	10	1.8 ± 0.4
G– sepsis	10	4.4 ± 0.6
G– sepsis + Xiangdan 2.0 mL/kg	10	3.1 ± 0.6
G– sepsis + Xiangdan 2.5 mL/kg	10	2.4 ± 0.5
G– sepsis + Xiangdan 5.0 mL/kg	10	1.7 ± 0.3

### 3.3 Inflammatory cytokine levels

**Figure 4:**
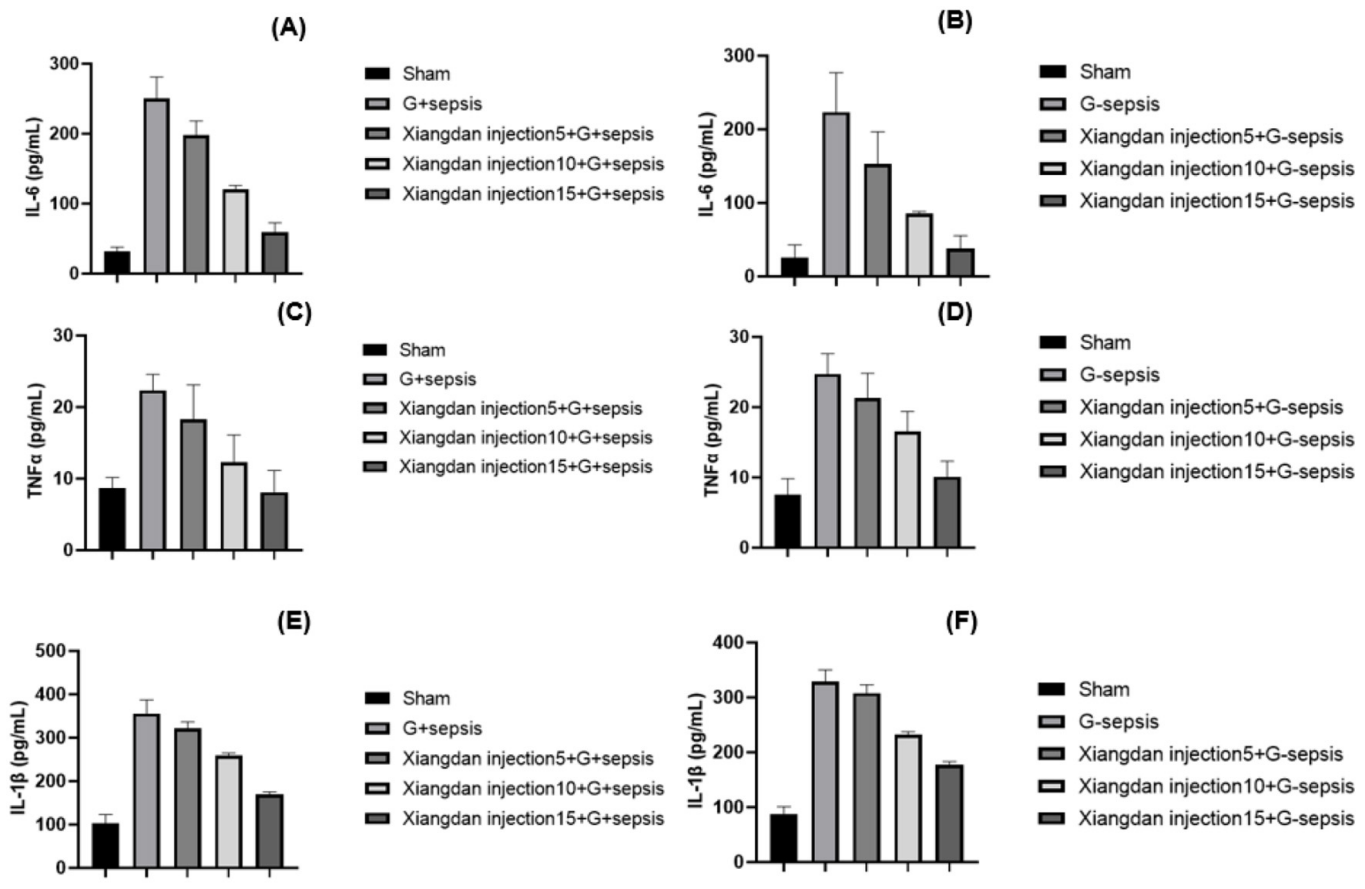
Markers of inflammatory cytokines in mice caused by G+/G– bacteria different concentrations of Xiangdan injection intervention. Bar chart with error bard (mean ± SD) **(A)** IL-6 levels in each group of G+ mice, *n* = 5; **(B)** IL-6 levels in each group of G– mice, *n* = 5; **(C)** TNF α levels in each group of G+ mice, *n* = 5; **(D)** TNF α levels in each group of G– mice, *n* = 5; **(D)** TNF α levels in each group of G– mice, *n* = 5; **(D)** TNF α levels in each group of G- mice, *n* = 5; **(E)** IL-1β levels in each group of G+ mice, *n* = 5; **(F)** IL-1β levels in each group of G– mice, *n* = 5. G+ sepsis is the Staphylococcus aureus sepsis group; G– sepsis is the Escherichia coli sepsis group.

**Figure 5:**
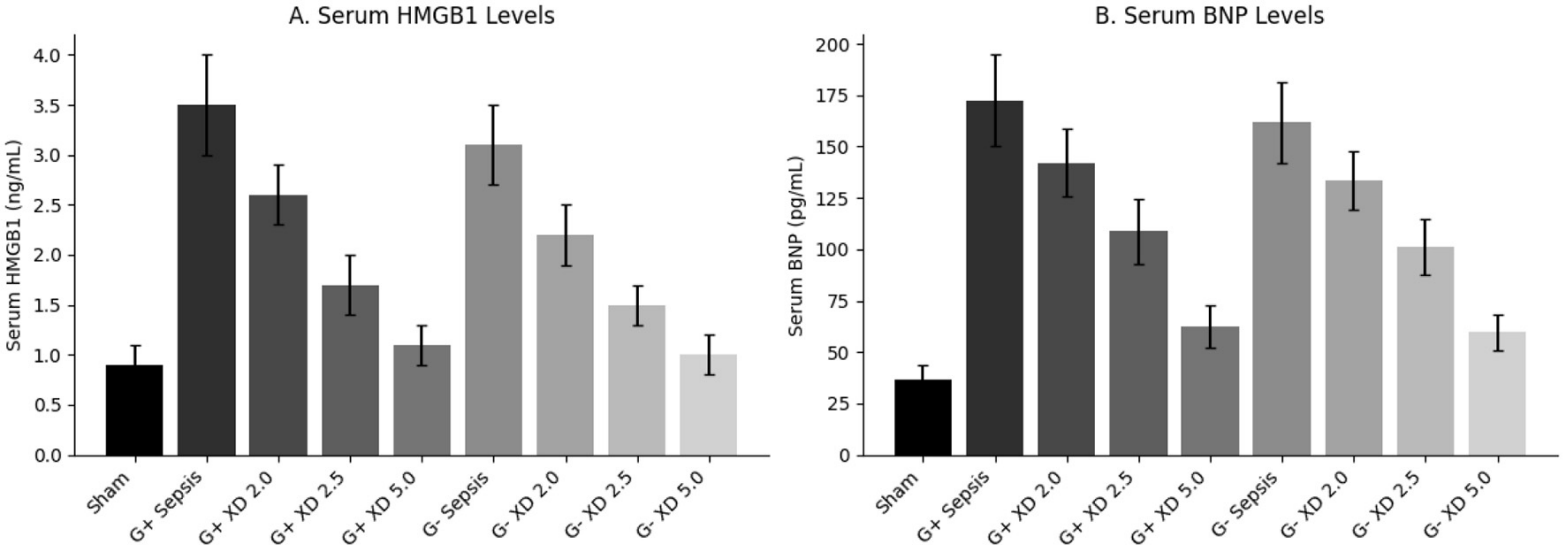
Serum HMGB1 and BNP Levels in Each Group. **(A, B)** Display group means (± SD) for serum HMGB1 and BNP, respectively. Xiangdan injection reduced sepsis-induced elevations in both biomarkers in a dose-dependent fashion.

Serum levels of IL-6, TNF-α, IL-1β, and HMGB1 were markedly elevated in sepsis groups compared to Sham (*P* < 0.001). Xiangdan injection significantly reduced these inflammatory markers in a dose-dependent manner (*P* < 0.05), with cytokine levels in the 5.0 mL/kg group approaching those in the Sham group (Table 5; Figures 4, 5).

**Table 5:** Serum inflammatory cytokine levels in each group.

**Group**	***n***	**IL-6 (pg/mL) (mean ±SD)**	**TNF-α (pg/mL) (mean ±SD)**	**IL-1β (pg/mL) (mean ±SD)**	**HMGB1 (ng/mL) (mean ±SD)**
Sham	10	15.4 ± 3.1	19.7 ± 3.2	12.6 ± 2.8	0.9 ± 0.2
G+ sepsis	10	102.7 ± 13.6	98.5 ± 12.5	68.1 ± 8.9	3.5 ± 0.5
G+ sepsis + Xiangdan 2.0 mL/kg	10	73.2 ± 8.4	70.3 ± 9.8	50.2 ± 7.1	2.6 ± 0.3
G+ sepsis + Xiangdan 2.5 mL/kg	10	52.5 ± 7.9	51.9 ± 7.6	36.7 ± 5.5	1.7 ± 0.3
G+ sepsis + Xiangdan 5.0 mL/kg	10	35.8 ± 5.2	32.1 ± 5.3	21.6 ± 3.8	1.1 ± 0.2
G– sepsis	10	94.8 ± 10.2	93.3 ± 10.6	62.3 ± 7.7	3.1 ± 0.4
G– sepsis + Xiangdan 2.0 mL/kg	10	69.7 ± 7.9	67.8 ± 7.2	45.2 ± 6.2	2.2 ± 0.3
G– sepsis + Xiangdan 2.5 mL/kg	10	47.9 ± 6.3	48.1 ± 6.0	30.8 ± 4.8	1.5 ± 0.2
G– sepsis + Xiangdan 5.0 mL/kg	10	33.2 ± 4.6	28.7 ± 3.9	19.8 ± 2.7	1.0 ± 0.2

### 3.4 Myocardial injury and heart failure markers

**Figure 6:**
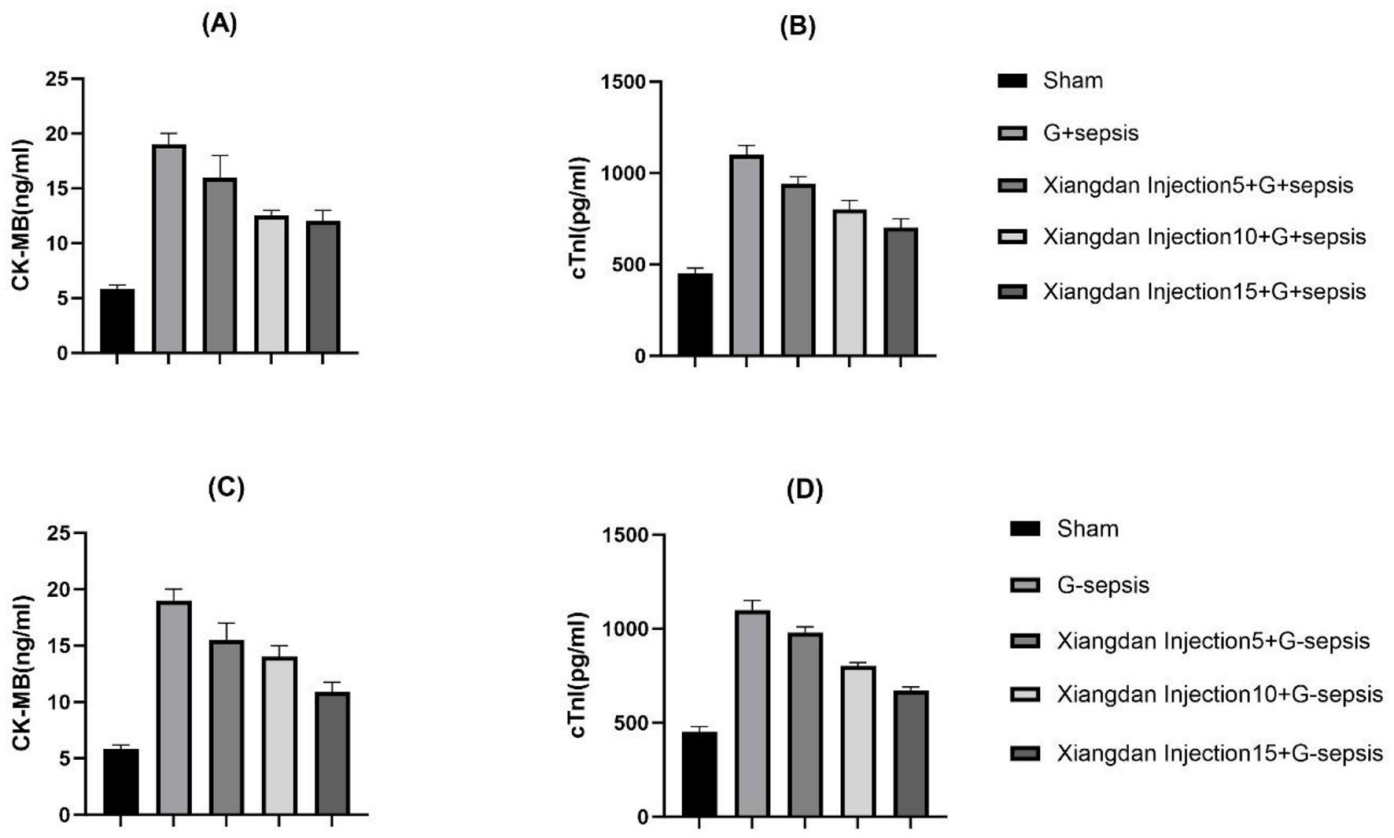
Markers of myocardial injury in mice caused by G+/G– bacteria different concentrations of Xiangdan injection intervention. Bar charts with error bars **(A)** Serum CK - MB levels in each group of G+ mice, *n* = 5; **(B)** Serum cTnI levels in each group of G+ mice, *n* = 5; **(C)** Serum CK - MB levels in each group of G– mice, *n* = 5; **(D)** Serum cTnI levels in each group of G– mice, *n* = 5; G+ sepsis is the *Staphylococcus aureus* sepsis group; G - sepsis is the *Escherichia coli* sepsis group.

Levels of cardiac biomarkers BNP, CK-MB, and cTnI were significantly increased in sepsis groups compared to Sham (*P* < 0.001), indicating myocardial damage. Xiangdan injection treatment significantly decreased levels of all three markers in a dose-dependent fashion (*P* < 0.05). At the highest dose (5.0 mL/kg), biomarker levels were substantially reduced and approached baseline (Table 6; Figure 6).

**Table 6:** Serum cardiac injury biomarkers in each group.

**Group**	***n***	**BNP (pg/mL) (mean ±SD)**	**CK-MB (U/L) (mean ±SD)**	**cTnI (ng/mL) (mean ±SD)**
Sham	10	36.7 ± 6.8	98.1 ± 13.5	0.19 ± 0.04
G+ sepsis	10	172.5 ± 22.1	398.7 ± 45.2	1.12 ± 0.19
G+ sepsis + Xiangdan 2.0 mL/kg	10	142.1 ± 16.3	335.2 ± 40.1	0.91 ± 0.13
G+ sepsis + Xiangdan 2.5 mL/kg	10	108.8 ± 15.9	272.6 ± 31.8	0.61 ± 0.09
G+ sepsis + Xiangdan 5.0 mL/kg	10	62.2 ± 10.3	147.3 ± 19.4	0.29 ± 0.06
G– sepsis	10	161.6 ± 19.5	377.6 ± 41.3	1.05 ± 0.16
G– sepsis + Xiangdan 2.0 mL/kg	10	133.3 ± 14.1	314.7 ± 28.2	0.85 ± 0.11
G– Sepsis + Xiangdan 2.5 mL/kg	10	101.1 ± 13.5	249.9 ± 24.6	0.55 ± 0.08
G– sepsis + Xiangdan 5.0 mL/kg	10	59.5 ± 8.7	140.2 ± 16.7	0.25 ± 0.04

### 3.5 Mitochondrial structural integrity

**Figure 7:**
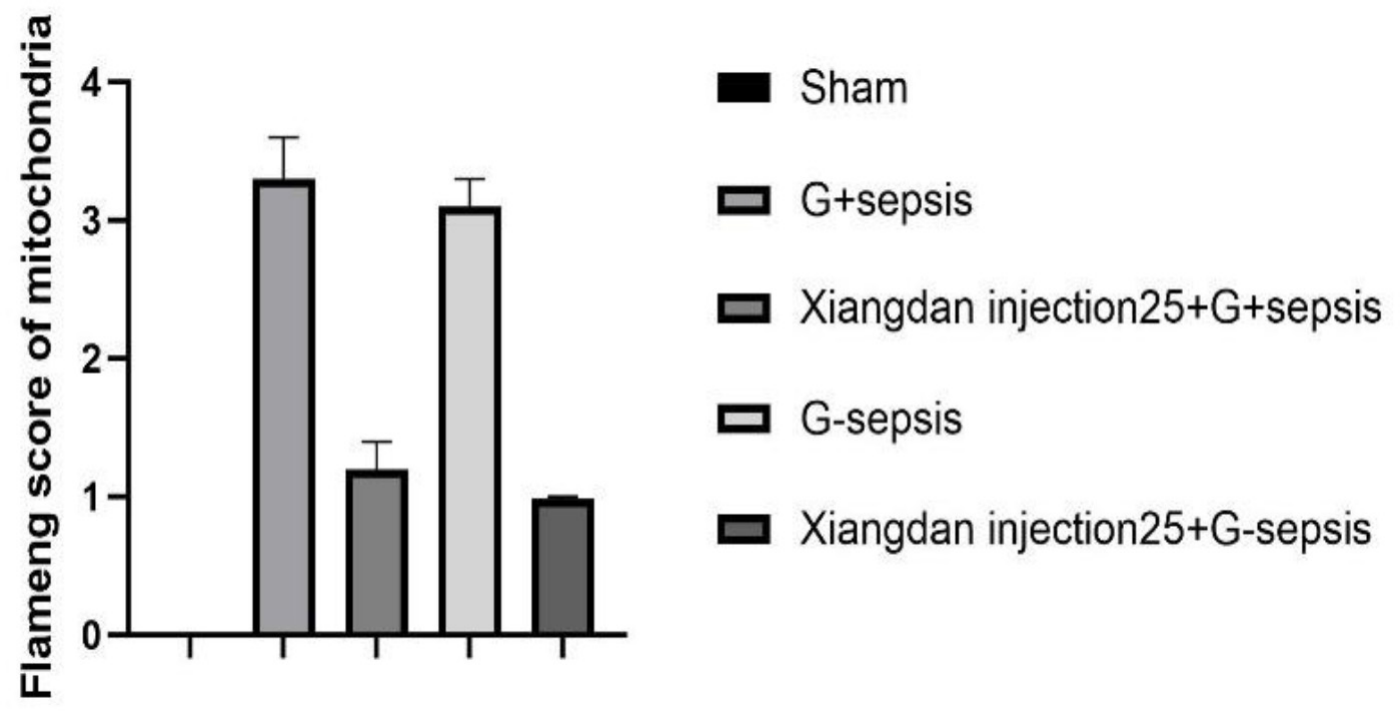
Mitochondrial injury score in septic mice caused by Xiangdan injection intervention with different strains. Myocardial mitochondrial changes and scores in rats of each group, *n* = 5. The G+ sepsis group is the Staphylococcus aureus sepsis group, and the G– sepsis group is the *Escherichia coli* sepsis group.

**Figure 8:**
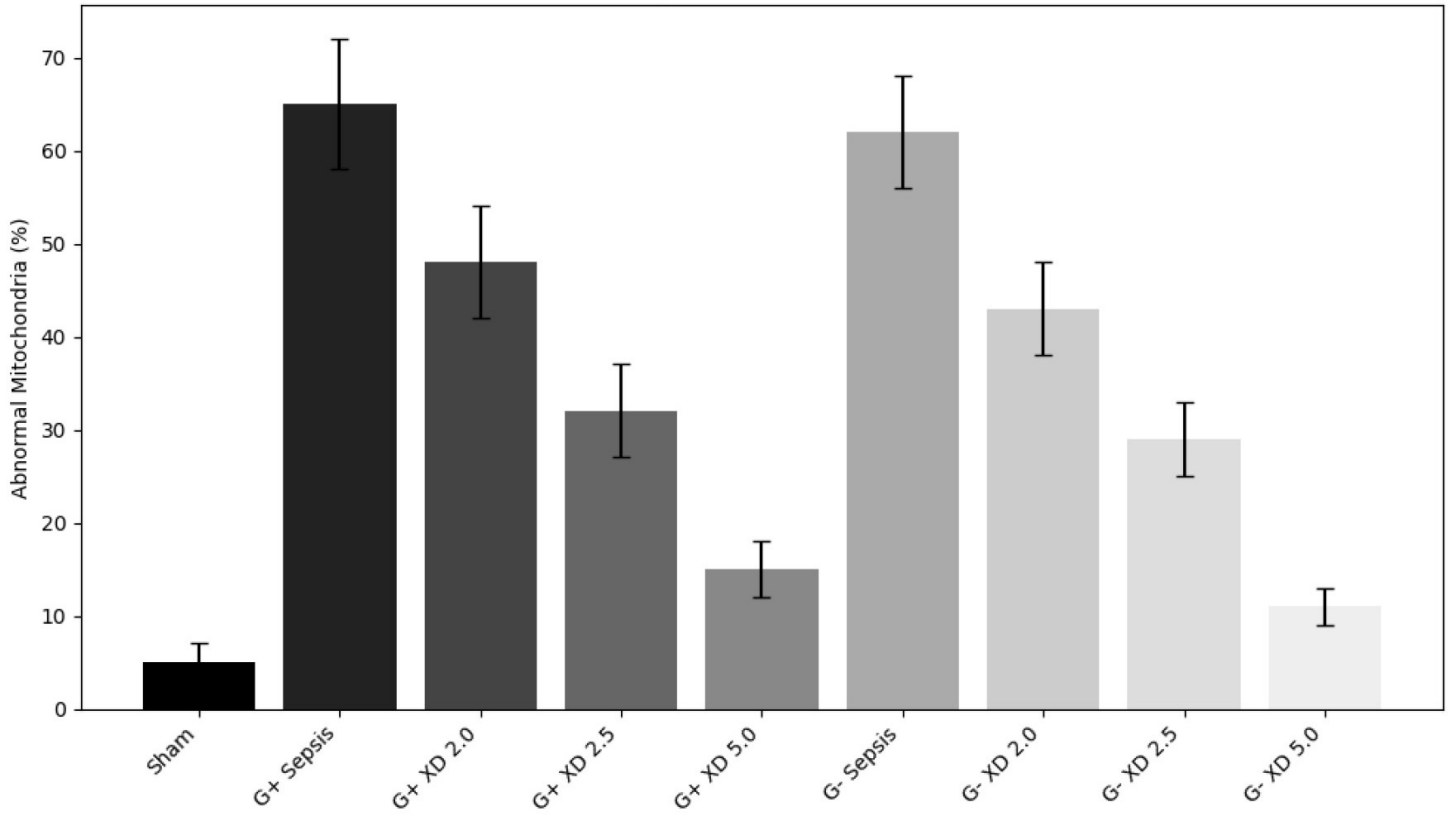
Quantitative analysis of mitochondrial abnormalities. Bar chart displays the percentage of abnormal mitochondria in myocardial tissue across all experimental groups. Xiangdan injection resulted in a dose-dependent reduction in mitochondrial abnormalities compared to untreated sepsis groups.

Transmission electron microscopy revealed well-preserved mitochondrial morphology in the Sham group, with intact double membranes and clear cristae. In contrast, mitochondria from sepsis groups displayed fragmentation, cristae dissolution, and vacuolar changes. Xiangdan injection significantly improved mitochondrial integrity, as reflected by reduced Flameng scores in a dose-dependent manner (*P* < 0.05). Mitochondria in the 5.0 mL/kg groups exhibited near-normal morphology (Table 7; Figures 7, 8).

**Table 7:** Mitochondrial Flameng scores in each group.

**Group**	***n***	**Flameng score (mean ±SD)**
Sham	10	0.8 ± 0.2
G+ sepsis	10	3.9 ± 0.5
G+ sepsis + Xiangdan 2.0 mL/kg	10	2.9 ± 0.4
G+ sepsis + Xiangdan 2.5 mL/kg	10	2.2 ± 0.3
G+ sepsis + Xiangdan 5.0 mL/kg	10	1.5 ± 0.4
G– sepsis	10	3.7 ± 0.4
G– sepsis + Xiangdan 2.0 mL/kg	10	2.7 ± 0.3
G– sepsis + Xiangdan 2.5 mL/kg	10	2.0 ± 0.2
G– sepsis + Xiangdan 5.0 mL/kg	10	1.3 ± 0.2

### 3.6 Dose-response relationship

**Figure 9:**
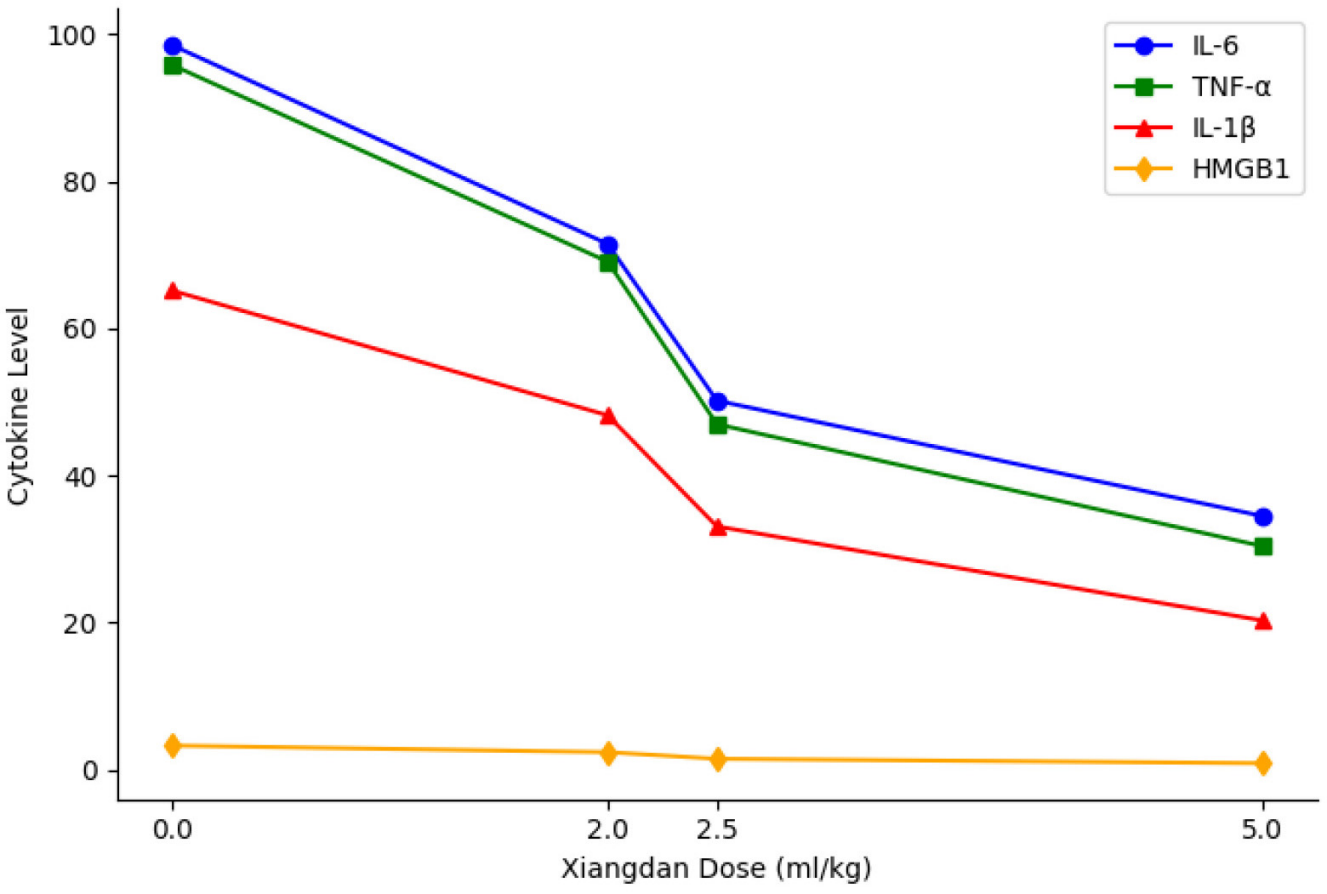
Dose-dependent reduction of inflammatory cytokines. Line graph demonstrates that increasing doses of Xiangdan injection resulted in progressive, dose-dependent reductions in IL-6, TNF-α, IL-1β, and HMGB1 levels in septic mice.

**Figure 10:**
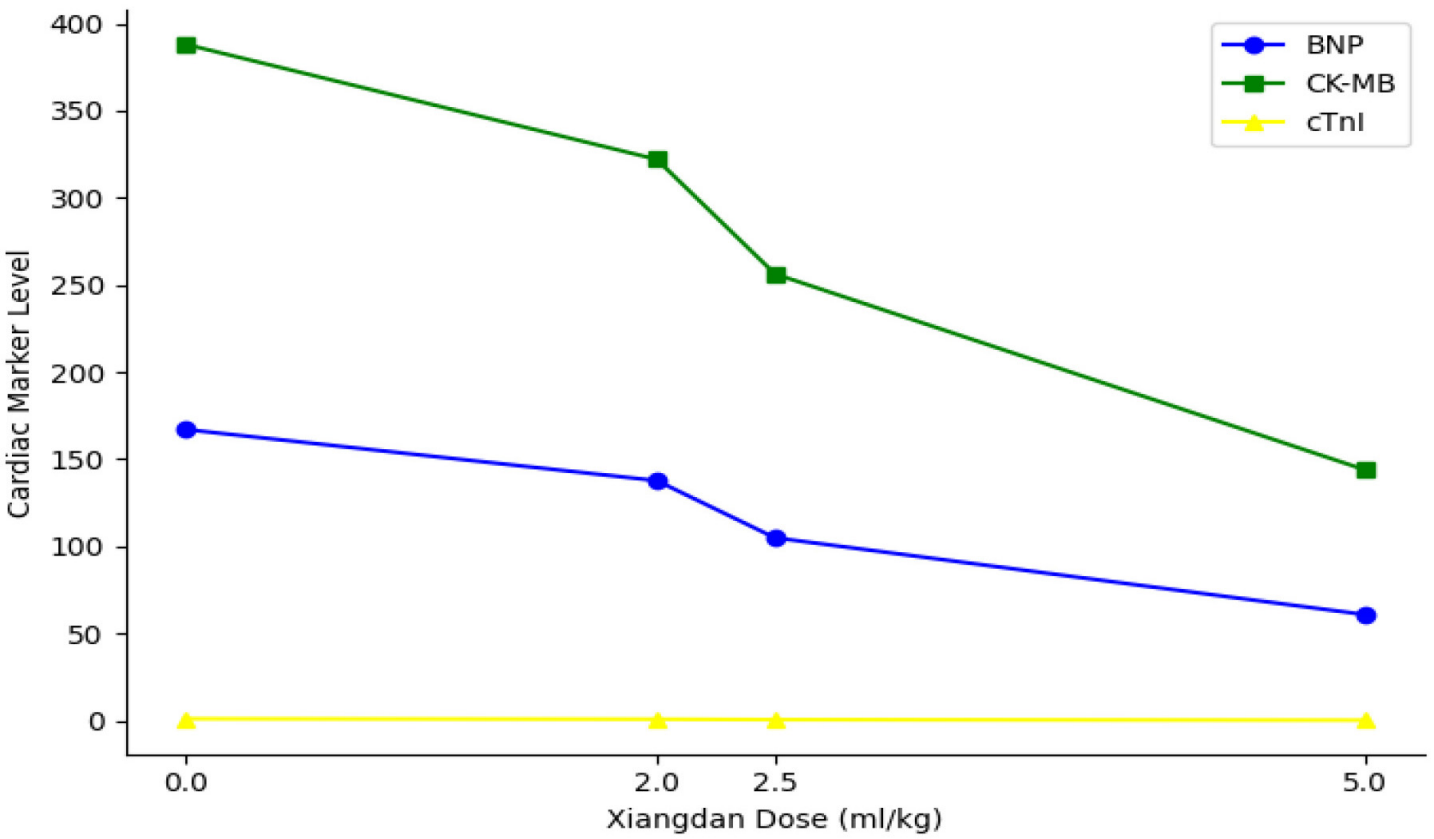
Dose-dependent reduction in cardiac injury markers. Line plot shows progressive, dose-dependent decreases in BNP, CK-MB, and cTnI levels with increasing doses of Xiangdan injection in septic mice.

**Figure 11:**
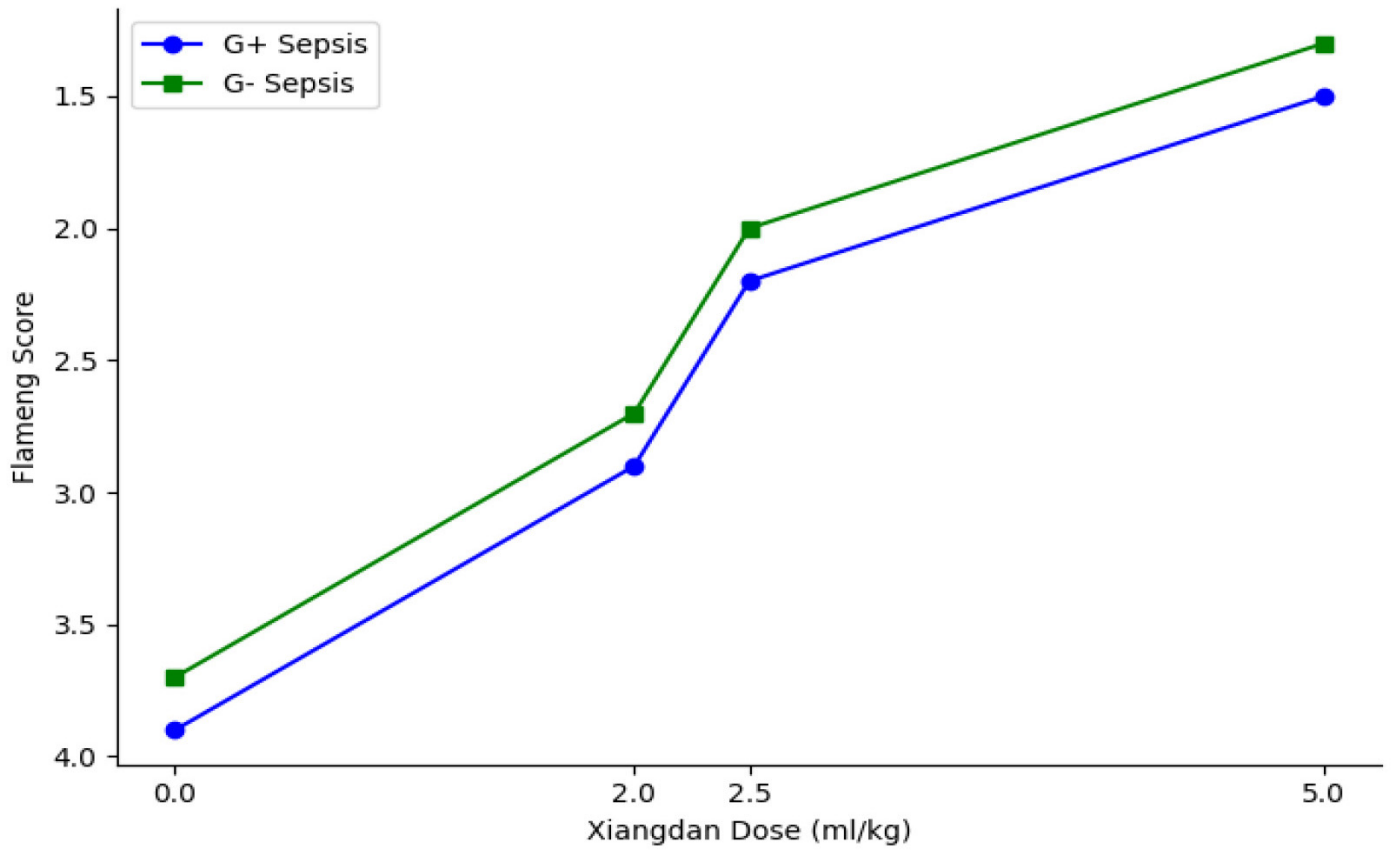
Dose-dependent preservation of mitochondrial integrity. Line plot demonstrates that increasing doses of Xiangdan injection lead to progressive, dose-dependent preservation of myocardial mitochondrial integrity (lower Flameng scores) in both G+ and G– sepsis models.

A clear dose-dependent effect of Xiangdan injection was observed across all key outcomes, including reductions in IL-6, BNP, and Flameng mitochondrial scores. These effects were consistent across both G+ and G– sepsis models, and summarized in Table 8 and Figures 9–11. Comparative analysis between G+ and G– sepsis groups revealed no statistically significant differences in most outcome measures (*P* > 0.05), suggesting consistent efficacy of Xiangdan injection regardless of bacterial strain (Table 9). A comprehensive summary of all outcomes by group is provided in Table 10.

**Table 8:** Dose-response analysis of Xiangdan injection effects.

**Dose (mL/kg)**	***n***	**IL-6 (pg/mL)**	**BNP (pg/mL)**	**Flameng score**
0 (sepsis)	20	98.6 ± 12.0	167.1 ± 20.2	3.8 ± 0.4
2.0	20	71.5 ± 8.2	137.7 ± 14.2	2.8 ± 0.3
2.5	20	50.2 ± 7.0	104.9 ± 13.2	2.1 ± 0.2
5.0	20	34.5 ± 4.3	60.8 ± 8.5	1.4 ± 0.3

**Table 9:** Comparison of outcome measures between G+ and G– sepsis groups.

**Parameter**	**G+ sepsis (mean ±SD)**	**G– sepsis (mean ±SD)**	***p*-value**
IL-6 (pg/mL)	102.7 ± 13.6	94.8 ± 10.2	0.17
BNP (pg/mL)	172.5 ± 22.1	161.6 ± 19.5	0.28
Flameng score	3.9 ± 0.5	3.7 ± 0.4	0.22

Table 10Summary of all major outcome measures by group.

**Table:** 

**A. Survival and histopathological scores**		
**Group**	**Survival (%)**	**Histopathological score (mean** ±**SD)**
Sham	100	0.7 ± 0.2
G+ sepsis	60	4.6 ± 0.7
G+ sepsis + Xiangdan 2.0	70	3.4 ± 0.5
G+ sepsis + Xiangdan 2.5	80	2.7 ± 0.4
G+ sepsis + Xiangdan 5.0	100	1.8 ± 0.4
G– sepsis	70	4.4 ± 0.6
G– sepsis + Xiangdan 2.0	80	3.1 ± 0.6
G– sepsis + Xiangdan 2.5	90	2.4 ± 0.5
G– sepsis + Xiangdan 5.0	100	1.7 ± 0.3

**Table:** 

**B. Serum inflammatory cytokine levels**				
**Group**	**IL-6 (pg/mL)**	**TNF-**α **(pg/mL)**	**IL-1**β **(pg/mL)**	**HMGB1 (ng/mL)**
Sham	15.4 ± 3.1	19.7 ± 3.2	12.6 ± 2.8	0.9 ± 0.2
G+ sepsis	102.7 ± 13.6	98.5 ± 12.5	68.1 ± 8.9	3.5 ± 0.5
G+ sepsis + Xiangdan 2.0	73.2 ± 8.4	70.3 ± 9.8	50.2 ± 7.1	2.6 ± 0.3
G+ sepsis + Xiangdan 2.5	52.5 ± 7.9	51.9 ± 7.6	36.7 ± 5.5	1.7 ± 0.3
G+ sepsis + Xiangdan 5.0	35.8 ± 5.2	32.1 ± 5.3	21.6 ± 3.8	1.1 ± 0.2
G– sepsis	94.8 ± 10.2	93.3 ± 10.6	62.3 ± 7.7	3.1 ± 0.4
G– sepsis + Xiangdan 2.0	69.7 ± 7.9	67.8 ± 7.2	45.2 ± 6.2	2.2 ± 0.3
G– sepsis + Xiangdan 2.5	47.9 ± 6.3	48.1 ± 6.0	30.8 ± 4.8	1.5 ± 0.2
G– sepsis + Xiangdan 5.0	33.2 ± 4.6	28.7 ± 3.9	19.8 ± 2.7	1.0 ± 0.2
**C. Cardiac biomarkers and mitochondrial integrity**				
**Group**	**BNP (pg/mL)**	**CK-MB (U/L)**	**cTnI (ng/mL)**	**Flameng score (mean** ±**SD)**
Sham	36.7 ± 6.8	98.1 ± 13.5	0.19 ± 0.04	0.8 ± 0.2
G+ sepsis	172.5 ± 22.1	398.7 ± 45.2	1.12 ± 0.19	3.9 ± 0.5
G+ sepsis + Xiangdan 2.0	142.1 ± 16.3	335.2 ± 40.1	0.91 ± 0.13	2.9 ± 0.4
G+ sepsis + Xiangdan 2.5	108.8 ± 15.9	272.6 ± 31.8	0.61 ± 0.09	2.2 ± 0.3
G+ sepsis + Xiangdan 5.0	62.2 ± 10.3	147.3 ± 19.4	0.29 ± 0.06	1.5 ± 0.4
G– sepsis	161.6 ± 19.5	377.6 ± 41.3	1.05 ± 0.16	3.7 ± 0.4
G– sepsis + Xiangdan 2.0	133.3 ± 14.1	314.7 ± 28.2	0.85 ± 0.11	2.7 ± 0.3
G– sepsis + Xiangdan 2.5	101.1 ± 13.5	249.9 ± 24.6	0.55 ± 0.08	2.0 ± 0.2
G– sepsis + Xiangdan 5.0	59.5 ± 8.7	140.2 ± 16.7	0.25 ± 0.04	1.3 ± 0.2

## 4 Discussion

This study demonstrates that Xiangdan injection exerts significant, dose-dependent cardioprotective effects in murine models of sepsis-induced myocardial injury (SIMI) caused by both *S. aureus* (Gram-positive) and *E. coli* (Gram-negative) infections. Key findings include improved 12-h survival, reduced myocardial pathological damage, decreased serum inflammatory cytokines (IL-6, TNF-α, IL-1β, HMGB1), lowered cardiac injury biomarkers (BNP, CK-MB, cTnI), and preservation of myocardial mitochondrial structure. These protective effects were consistent across bacterial infection models, highlighting Xiangdan injection's broad preclinical potential.

Our results align with previous research on Traditional Chinese Medicine (TCM) injections in sepsis, particularly Xuebijing (XBJ), which has been shown to reduce mortality, systemic inflammation, and organ injury in both preclinical and clinical settings (13–15, 21–23). The present study extends this evidence by demonstrating that Xiangdan injection confers comparable cardioprotective effects in both Gram-positive and Gram-negative sepsis models, a distinction not fully explored previously. Reductions in serum IL-6, TNF-α, IL-1β, and HMGB1 after Xiangdan injection administration indicate potent anti-inflammatory activity, consistent with prior reports that related formulations can modulate TLR4/NF-κB and JAK2/STAT3 signaling (7–12, 17–19). In parallel, the observed improvements in mitochondrial morphology (lower Flameng scores) support previous observations that mitochondrial dysfunction is a hallmark of SIMI and a major contributor to energy failure in the septic heart (16–19, 25–28).

Because we did not directly measure TLR4, NF-κB, JAK2, STAT3, or their phosphorylation states, we have tempered our interpretation to state that our findings are consistent with, but do not directly prove, modulation of these pathways. Future studies should incorporate Western blotting or immunohistochemistry for TLR4, p-p65, p-IκBα, JAK2, and p-STAT3 with appropriate loading controls to confirm these intracellular mechanisms (7–12, 17–19).

The experimental design employed a prophylactic plus early post-insult regimen (12 h before and immediately after bacterial challenge). While this approach allowed us to examine potential preventive effects, it limits clinical generalizability, since most sepsis therapy occurs after diagnosis. A logical next step is a strictly post-insult dosing study to model real-world therapeutic intervention and evaluate whether Xiangdan injection retains efficacy when administered after sepsis onset (28–32). We also used only male C57BL/6 mice. Sex-based differences in immune responses and cardiac injury have been documented in sepsis (28, 29), so future work should include female mice and other strains to improve generalizability. Finally, the assessment was limited to the acute phase (12 h) after sepsis induction; longer-term studies are needed to evaluate sustained myocardial protection and recovery (27, 33–36).

Despite these limitations, our study provides new ultrastructural evidence of mitochondrial protection by Xiangdan injection in the context of SIMI. This finding is consistent with earlier work showing that preservation of mitochondrial cristae and membranes correlates with improved cardiac function and survival in sepsis models (25–28). Together with the dose-dependent reduction of cytokines and cardiac injury biomarkers, these data support Xiangdan injection's translational potential as an adjunctive therapy for sepsis-induced myocardial injury. As the experiment was designed with a prespecified 12-h humane endpoint, survival reflects early-phase mortality rather than long-term outcomes. Future studies will extend follow-up and evaluate long-term survival and cardiac function.

Xiangdan injection is formulated for intravenous use clinically but was administered orally by gavage in our study, differences in absorption and bioavailability must be considered when extrapolating our findings to clinical practice. We have provided the manufacturer's HPLC profile and calculated active compound exposures to facilitate cross-study comparison. Future work should directly compare intravenous and oral administration in animal models and incorporate pharmacokinetic measurements to better inform clinical translation.

The scientific and clinical implications of these findings are considerable. Demonstrating consistent cardioprotection and mitochondrial preservation by Xiangdan injection in both Gram-positive and Gram-negative sepsis models underscores its translational potential as an adjunctive therapy. Beyond confirming dose-dependent benefits, these results broaden our mechanistic understanding of how Traditional Chinese Medicine (TCM) interventions may modulate inflammatory and mitochondrial pathways in sepsis. Collectively, they provide a strong foundation for future preclinical and clinical investigations.

Future research should extend these findings in several important directions. Longitudinal studies evaluating cardiac function, structural remodeling, and long-term survival after sepsis and TCM therapy are essential to determine whether the early benefits observed here translate into durable clinical outcomes. Mechanistic investigations employing knockout or transgenic models would help to pinpoint specific signaling mediators and clarify the causal pathways involved. In parallel, fractionating and testing the individual components of Xiangdan injection could identify the most active constituents responsible for myocardial protection, improving standardization and dosing precision.

Clinical translation will also require trials in diverse patient populations, including individuals with varying sepsis etiologies, comorbidities, and across different age and sex groups, to ensure generalizability and identify subgroups most likely to benefit. Finally, rigorous evaluation of potential interactions between Xiangdan injection and conventional treatments, such as antibiotics and vasoactive agents will be critical to ensure safety and to optimize combined therapeutic protocols. Together, these avenues of research will help advance Xiangdan injection from experimental use toward evidence-based integration into sepsis care.

Our dose selection was guided by published pharmacological evidence and preliminary tolerability data, not by formal GLP toxicology studies. Although no adverse events were observed even at the highest dose, future work should include formal pharmacokinetic and toxicity assessments to refine optimal dosing and better inform clinical translation.

This study used only male C57BL/6 mice to reduce hormonal variability and experimental heterogeneity. While this design facilitates detection of treatment effects in early-phase studies, it does not account for potential sex-based differences in immune and cardiovascular responses to sepsis. Future studies should include both male and female animals to capture possible sex-specific effects of Xiangdan injection and improve translational validity.

While our findings are consistent with modulation of TLR4/NF-κB and JAK2/STAT3 signaling pathways, these mechanisms were not directly confirmed. Future studies will incorporate pathway-specific inhibitors and molecular analyses such as Western blotting and immunohistochemistry to validate these pathways and elucidate the precise molecular targets of Xiangdan injection.

Because this study used a prophylactic plus early intervention dosing regimen, it does not fully replicate clinical treatment conditions, where therapy typically begins after sepsis diagnosis. Future studies should implement strictly post-insult dosing protocols to better model clinical administration. Furthermore, formal pharmacokinetic and toxicological studies are needed to evaluate bioavailability, dosing dynamics, and potential adverse effects. Although no acute adverse events were observed in the present work, such assessments will be critical to optimize safety and translational applicability of Xiangdan injection.

## 5 Conclusion

This study shows that Xiangdan injection provides powerful, dose-dependent protection against sepsis-induced myocardial injury in male mice. It improved short-term survival, blunted pro-inflammatory cytokines, decreased cardiac injury biomarkers, and preserved myocardial mitochondrial ultrastructure in both Gram-positive and Gram-negative sepsis models. Taken together, these findings imply that Xiangdan injection acts on upstream inflammatory and mitochondrial pathways; in line with previous evidence, our results are consistent with but do not directly confirm TLR4/NF-κB and JAK2/STAT3 pathway modulation. Future work will (i) directly interrogate these intracellular pathways using protein assays, (ii) test strictly post-insult dosing schedules, (iii) include both sexes and diverse strains, (iv) evaluate long-term cardiac outcomes to confirm and extend these mechanistic insights.

## Data Availability

The original contributions presented in the study are included in the article/Supplementary material, further inquiries can be directed to the corresponding author.
