# Supplementary material for: Attenuation of sepsis-induced myocardial injury by Xiangdan injection via mitochondrial protection and inflammation suppression in mice
Source: Front Med (Lausanne). 2025 Nov 7;12:1669474. doi: 10.3389/fmed.2025.1669474 (PMC12634624; doi:10.3389/fmed.2025.1669474)
Supplement: Supplementary file 1 [file Data_Sheet_1.docx]

Supplemental Table S1. Two-way ANOVA results for primary outcomes (IL-6, BNP, Flameng score) showing effects of bacterial type, dose, and their interaction.

| **Outcome** | **Factor** | **F value** | **p value** | **Partial η² (Effect Size)** | **95% CI for Effect Size** |
| --- | --- | --- | --- | --- | --- |
| IL-6 (pg/mL) | Bacterial Type | 1.92 | 0.17 | 0.03 | 0.00–0.08 |
| BNP (pg/mL) | Bacterial Type | 2.04 | 0.14 | 0.04 | 0.00–0.09 |
| Flameng Score | Bacterial Type | 1.75 | 0.19 | 0.03 | 0.00–0.07 |
| IL-6 (pg/mL) | Dose | 85.7 | <0.001 | 0.72 | 0.60–0.80 |
| BNP (pg/mL) | Dose | 102.4 | <0.001 | 0.78 | 0.65–0.85 |
| Flameng Score | Dose | 97.6 | <0.001 | 0.75 | 0.63–0.82 |
| IL-6 (pg/mL) | Bacterial Type × Dose | 0.88 | 0.42 | 0.01 | 0.00–0.05 |
| BNP (pg/mL) | Bacterial Type × Dose | 1.12 | 0.36 | 0.02 | 0.00–0.06 |
| Flameng Score | Bacterial Type × Dose | 0.95 | 0.39 | 0.01 | 0.00–0.05 |

Supplemental Table S2. Estimated exposure to major active constituents of Xiangdan injection at each oral dose level.

| **Xiangdan Dose (mL/kg)** | **Tanshinone IIA (mg/kg)** | **Salvianolic Acid B (mg/kg)** | **Rosmarinic Acid (mg/kg)** |
| --- | --- | --- | --- |
| 2.0 | 0.54 | 2.06 | 1.30 |
| 2.5 | 0.68 | 2.58 | 1.63 |
| 5.0 | 1.35 | 5.15 | 3.25 |
